# Robust Photocatalytic Hydrogen Evolution Over a Conjugated Metal‐Organic Framework Heterojunction

**DOI:** 10.1002/advs.76912

**Published:** 2026-08-03

**Authors:** Chunzhe Wang, Yufei Shan, Ruobing Chu, Lingzhi Yang, Zhanning Liu, Xiaoli Zhang, Jian Tian

**Affiliations:** ^1^ State Key Laboratory of Disaster Prevention and Ecology Protection in Open‐pit Coal Mines Shandong Key Laboratory of Special Epoxy Resin School of Materials Science and Engineering Shandong University of Science and Technology Qingdao China; ^2^ Australian Research Council Centre of Excellence for Green Electrochemical Transformation of Carbon Dioxide Applied Chemistry and Environmental Science School of Science RMIT University Melbourne VIC Australia

**Keywords:** π–π interaction, conjugated MOF, heterostructure, photocatalysis

## Abstract

Efficient photocatalysis requires the synergistic integration of charge separation and directional charge transport, yet achieving these features within a single framework remains challenging. Here, we report a conjugated coordination polymer, Cd‐TMT (TMT = 1,3,5‐trimercaptotriazine), featuring an intrinsic molecular‐level charge transport network. The conjugated TMT ligands facilitate strong intra‐ligand charge transfer, while their ordered *π‐π* stacking along the [111] direction establishes a continuous pathway for directional electron migration, endowing Cd‐TMT with intrinsic photocatalytic activity for hydrogen evolution. To further promote charge separation, Cd‐TMT is integrated with the conductive metal‐organic framework Cu‐HHTP (HHTP = 2,3,6,7,10,11‐hexahydroxytriphenylene) to construct a heterostructure. Spectroscopic investigations and density functional theory calculations demonstrate that this architecture accelerates charge separation while retaining highly reductive electrons. As a result, the Cd‐TMT@Cu‐HHTP composite achieves a H_2_ evolution rate of 9692.83 µmol g^−1^ h^−1^, surpassing most reported MOF‐based photocatalysts. This work provides fundamental insights into molecular‐level charge transport and offers a viable strategy for designing high‐performance heterostructures for solar‐to‐fuel conversion.

## Graphical abstract

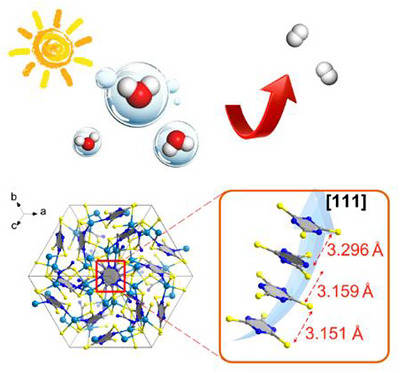

## 1 Introduction

Hydrogen is widely recognized as a clean energy carrier with great potential to facilitate the transition toward a sustainable energy future [1, 2, 3]. Among the various hydrogen production strategies, photocatalytic water splitting represents a particularly appealing solar‐to‐chemical energy conversion route and has attracted intense research attention [4, 5]. In this process, semiconductor photocatalysts absorb solar photons to excite electrons into the conduction band, generating highly reducing electrons that drive proton reduction to hydrogen. Consequently, the efficiency of this process is fundamentally governed by the properties of the photocatalyst. Since the pioneering discovery of TiO_2_ [6], diverse photocatalytic materials have been extensively explored, including oxides [7], sulfides [8], single‐atom catalysts [9], and polymers [10]. Despite these advances, a central bottleneck persists. In most conventional systems, photogenerated charge carriers migrate through intrinsically disordered and non‐directional pathways, leading to severe recombination losses and inefficient electron utilization. This fundamental limitation critically constrains photocatalytic performance. Therefore, the rational establishment of directional charge‐transport pathways has become a key design principle for highly efficient photocatalysts. To address this challenge, a variety of strategies have been developed, such as cocatalyst integration [11], heterostructure construction [12], and strain engineering [13]. Nevertheless, constructing well‐defined charge transport pathways at the molecular level remains highly challenging.

Metal‐organic frameworks (MOFs) and coordination polymers (CPs) are crystalline inorganic‐organic hybrids constructed from metal nodes and organic ligands. Benefiting from their high structural tunability, these materials have been recognized as ideal platforms for the rational design of targeted photocatalysts. Furthermore, their well‐defined crystal structures offer an excellent opportunity to gain atomic‐level insights into structure‐property relationships [14, 15]. For instance, Shi et al. constructed an ordered donor–acceptor (D–A) MOF via a mixed‐ligand strategy [16], where spatially separated donor and acceptor units promote charge separation and suppress recombination. In parallel, Eder et al. reported that COK‐47 exhibits pronounced ligand‐to‐metal charge transfer (LMCT) [17], driving electron migration from ligands to metal nodes and thereby enhancing charge separation. These studies highlight that the structural programmability of MOFs enables precise control over charge separation and transfer pathways, providing a foundation for efficient photocatalysis.

In this manuscript, the crystal structures and photocatalytic properties of Cd‐TMT (TMT = 1,3,5‐trimercaptotriazine) were investigated. Detailed structural analyses reveal that the conjugated TMT ligand enables pronounced intra‐ligand charge transfer (ILCT), while its ordered packing along the [111] direction facilitates efficient photoinduced electron transport. To further promote charge separation, a conductive MOF, Cu‐HHTP (HHTP = 2,3,6,7,10,11‐hexahydroxytriphenylene) [18], is integrated with Cd‐TMT to construct a heterostructure [19]. This architecture synergistically enhances charge separation and directional carrier migration. Remarkably, the optimized photocatalyst achieves a hydrogen evolution rate of 9692.83 µmol g^−1^ h^−1^.

## 2 Results and Discussions

**FIGURE 1:**
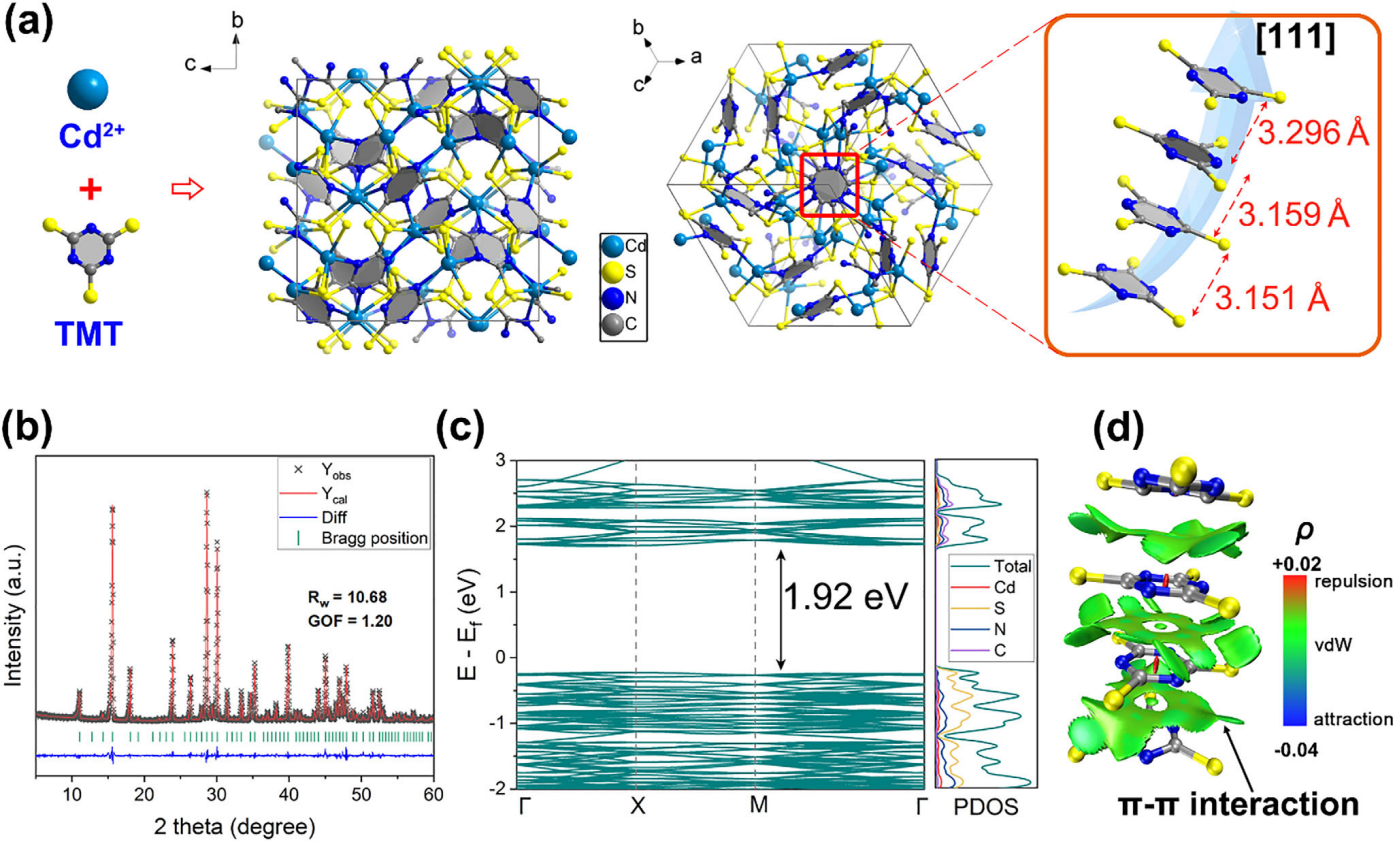
(a) Scheme of the crystal structure of Cd‐TMT and the existence of *π–π* interactions between adjacent TMT cores along the [111] direction. (b) Rietveld refinement patterns of Cd‐TMT. (c) DFT‐calculated band structures and projected density of states. (d) IRI analysis of the packed TMT cores.

The sample Cd‐TMT was synthesized via a facile co‐precipitation method. Specifically, aqueous solutions of Cd(NO_3_)_2_·4H_2_O and Na_3_TMT were mixed and stirred at room temperature for 24 h. Notably, although this compound had been previously synthesized and applied in photocatalytic hydrogen peroxide and hydrogen production more than a decade ago [20, 21, 22], its crystal structure was only recently resolved using continuous rotation electron diffraction [23]. This advancement enables a deeper understanding of its structure‐property relationships. To validate the simulated structural model, Rietveld refinements were performed. As shown in Figure 1b and Table S1, an excellent fit was achieved based on the reported structure (R_w_ = 10.68, GOF = 1.20), supporting the reliability of the model used for subsequent density functional theory calculations. As shown in Figure 1a, the Cd‐TMT crystallizes in a cubic phase with the space group of *Pa*‐3 and a formula of Cd_3_(TMT)_2_. Each Cd center adopts a pentacoordinate geometry, coordinated by three sulfur atoms and two nitrogen atoms. Notably, along the [111] direction, the TMT ligands are closely packed, with the intermolecular distance ranging from 3.151 to 3.296 Å, indicating the presence of strong *π*‐*π* interactions [24]. This is further corroborated by the interaction region indicator (IRI) analysis (Figure 1d) [25], which reveals pronounced green isosurfaces associated with noncovalent *π*‐*π* interactions. The electronic structure was further examined by band structure and projected density of states (PDOS) analyses. As shown in Figure 1c, the calculated band gap is ∼1.92 eV. The valence band maximum is primarily dominated by S atoms, whereas the conduction band minimum mainly originates from C and N atoms. These results unveil a characteristic intra‐ligand charge transfer (ILCT) excitation [26], involving an electron transition from S centers to the π* orbitals of the conjugated ligand. Such intrinsic spatial separation of the band‐edge states, coupled with the well‐defined *π*‐*π* stacking along the [111] direction, is expected to promote efficient charge separation and directional carrier transport, thereby establishing Cd‐TMT as a highly promising photocatalyst.

**FIGURE 2:**
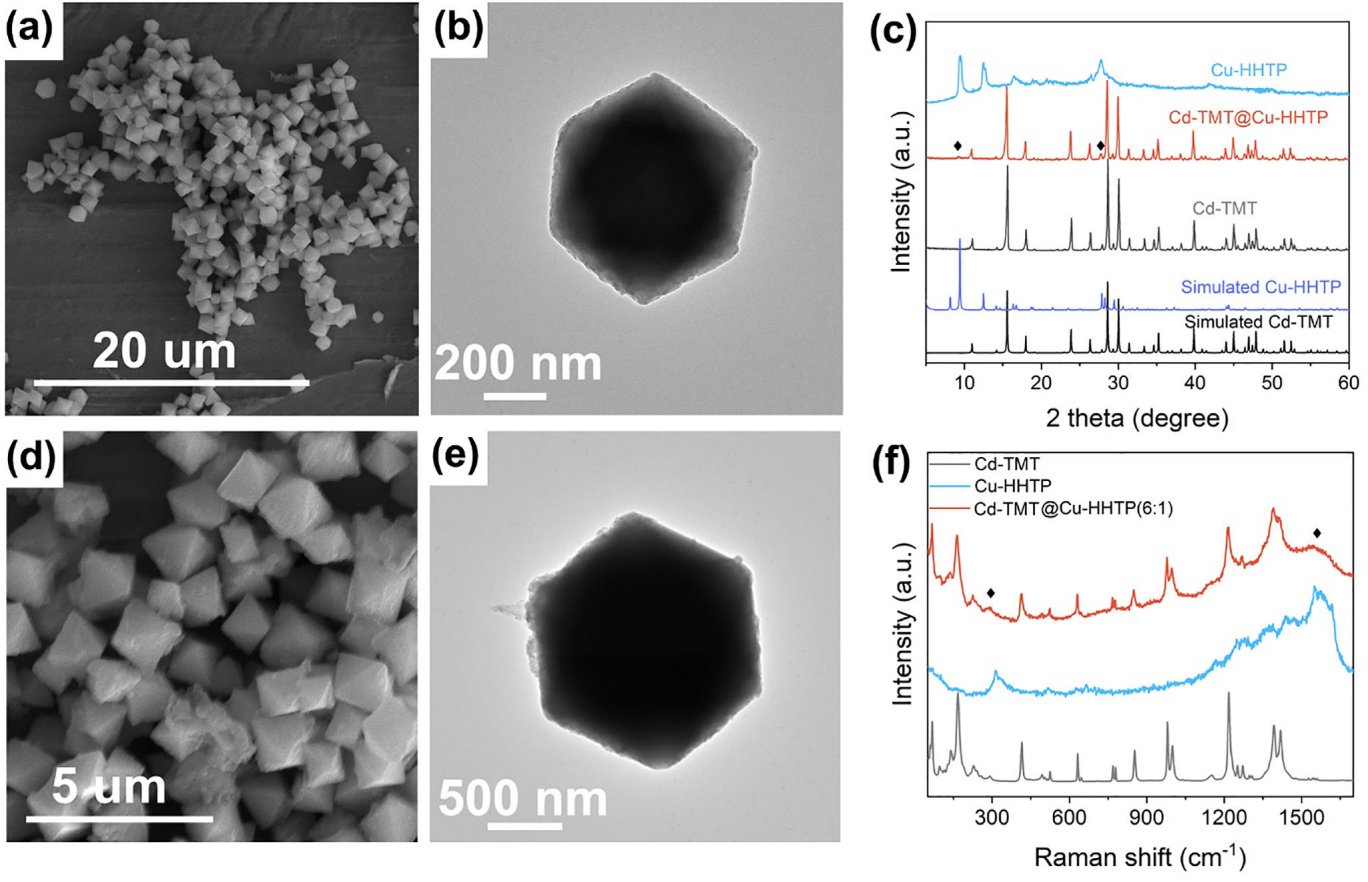
SEM and TEM images of pristine Cd‐TMT (a, b) and Cd‐TMT@Cu‐HHTP (6:1) (d, e). (c) PXRD patterns and (f) Raman spectra of the synthesized compounds.

Although Cd‐TMT exhibits a theoretically advantageous electronic structure for photocatalysis, its experimentally reported activity remains only moderate, suggesting that the intrinsic merits are hindered by inefficient charge transport and utilization. To bridge this gap, Cu‐HHTP, a representative electrically conductive MOF [18], was incorporated to construct a composite system. Scanning electron microscopy (SEM) and transmission electron microscopy (TEM) were utilized to investigate the morphology of the synthesized compounds. As shown in Figure 2a,b, pristine Cd‐TMT exhibits a uniform octahedral morphology with crystal sizes ranging from 400 to 600 nm. Based on its cubic crystal structure, such an octahedral morphology suggests that the exposed surfaces are likely dominated by (111) facets according to the Wulff construction. The corresponding selected‐area electron diffraction (SAED) pattern exhibits a characteristic sixfold symmetry (Figure S1), consistent with observation along the [111] zone axis, further supporting the crystallographic orientation of the crystals. After the incorporation of Cu‐HHTP, the octahedral morphology is well preserved, while the surface becomes slightly wrinkled (Figure 2d,e), suggesting the formation of a coating layer. Elemental mapping, together with the line‐scan analysis, further confirms the successful coating of Cu‐HHTP onto the Cd‐TMT surface (Figures S2,S3). The phase structures of the as‐prepared samples were examined by powder X‐ray diffraction (PXRD) analysis. As shown in Figure 2c, the diffraction patterns of pristine Cd‐TMT and Cu‐HHTP match well with their simulated counterparts, confirming their high purities. In the composite, the diffraction features are predominantly inherited from Cd‐TMT, while a characteristic reflection at 2θ = 9.5° can be indexed to the (220) plane of Cu‐HHTP [27], indicating successful incorporation without disrupting the host framework. Raman spectroscopy was further employed to probe the structural integration. Cd‐TMT exhibits sharp vibrational bands, in contrast to the broader features of Cu‐HHTP. Notably, the composite displays additional broadened bands centered at ∼300 and ∼1600 cm^−1^, which can be assigned to the vibrational modes of Cu‐HHTP. Collectively, the morphological analyses, PXRD patterns, and Raman spectra unambiguously confirm the successful construction of a Cd‐TMT@Cu‐HHTP heterostructure.

**FIGURE 3:**
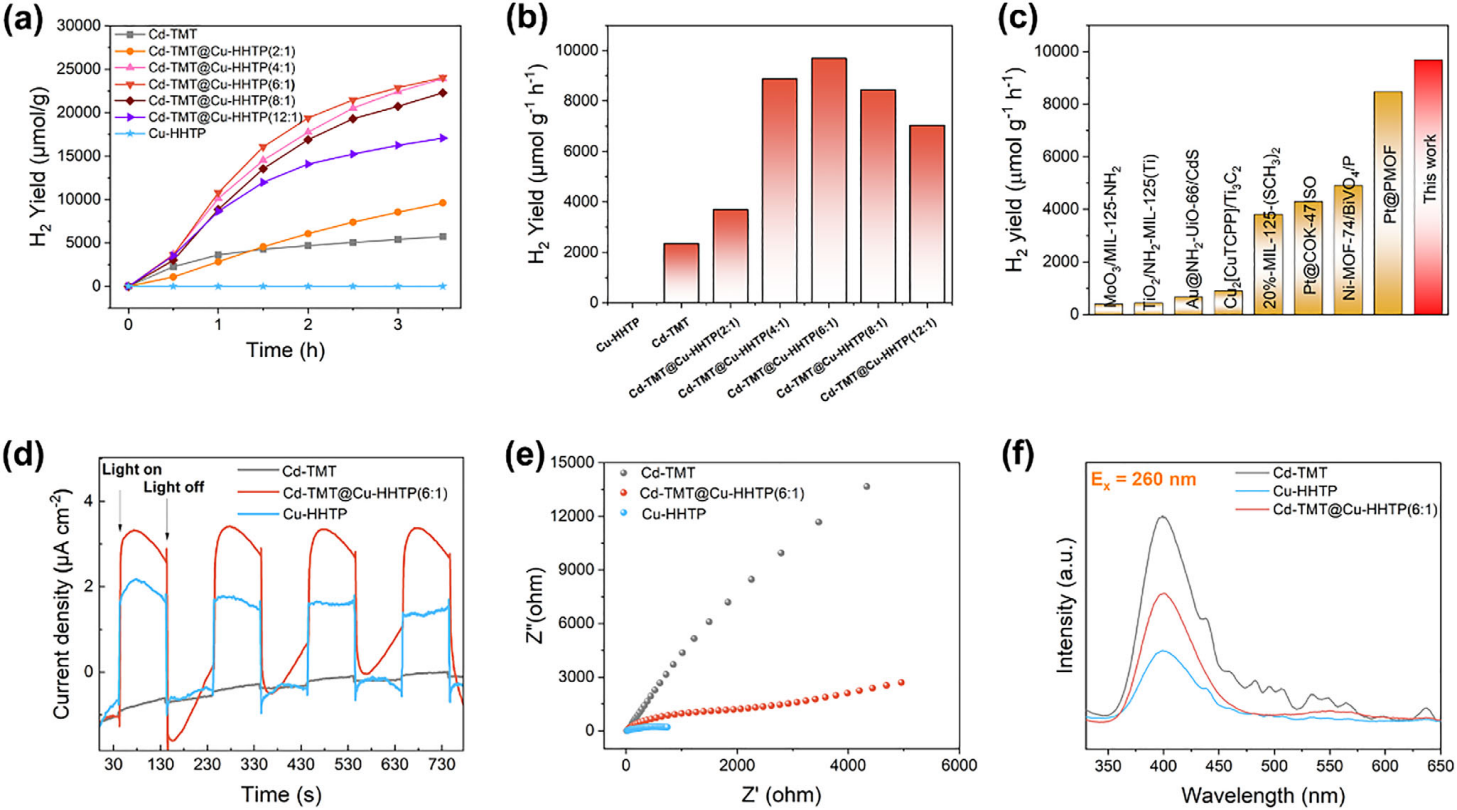
(a, b) Photocatalytic hydrogen evolution rate of Cd‐TMT, Cu‐TTHP, and their composites with different ratios. (c) Performance comparison of the composite Cd‐TMT@Cu‐HHTP (6:1) with reported MOF‐based photocatalysts. (d) Photocurrent response tests, (e) EIS spectra, and (f) PL spectra of Cd‐TMT, Cu‐TTHP, and Cd‐TMT@Cu‐HHTP (6:1).

The photocatalytic hydrogen evolution performances of the synthesized materials were evaluated under irradiation from a 300 W Xe lamp equipped with an AM 1.5 filter. For a clear comparison, the performances of pristine Cd‐TMT and Cu‐HHTP were measured under identical conditions. As shown in Figure 3a, pristine Cu‐HHTP shows negligible hydrogen production, while Cd‐TMT shows an average evolution rate of 2344.67 µmol g^−1^ h^−1^. Notably, the Cd‐TMT@Cu‐HHTP composites display significantly enhanced activities. As the ratio of Cd‐TMT to Cu‐HHTP increases, the average H_2_ yield rate follows a volcano‐like trend (Figure 3b), with Cd‐TMT@Cu‐HHTP (6:1) achieving the highest rate of 9692.83 µmol g^−1^ h^−1^, nearly four times higher than that of pristine Cd‐TMT and surpassing most of the reported MOF‐based photocatalysts (Figure 3c) [17, 28, 29, 30, 31, 32, 33, 34]. To investigate the stability of Cd‐TMT@Cu‐HHTP (6:1), three consecutive cycles of the photocatalytic reaction were carried out. No apparent decline in activity was observed (Figure S4). To reveal the mechanism for the outstanding performance of the heterostructure, the charge separation and migration process were investigated. Electrochemical impedance spectra (EIS) measurements showed that the Cd‐TMT@Cu‐HHTP (6:1) possesses a much smaller arc radius (Figure 3d), demonstrating reduced charge‐transfer resistance. The transient photocurrent measurements showed that the intensity follows the order of Cd‐TMT@Cu‐HHTP > Cd‐TMT > Cu‐HHTP (Figure 3e), which is consistent with the hydrogen evolution results. Moreover, the photoluminescence properties were also investigated. As shown in Figure 3f, under the same conditions, the emission intensity of Cd‐TMT@Cu‐HHTP is significantly lower than that of Cd‐TMT, indicating that the construction of a heterostructure can effectively inhibit carrier recombination.

**FIGURE 4:**
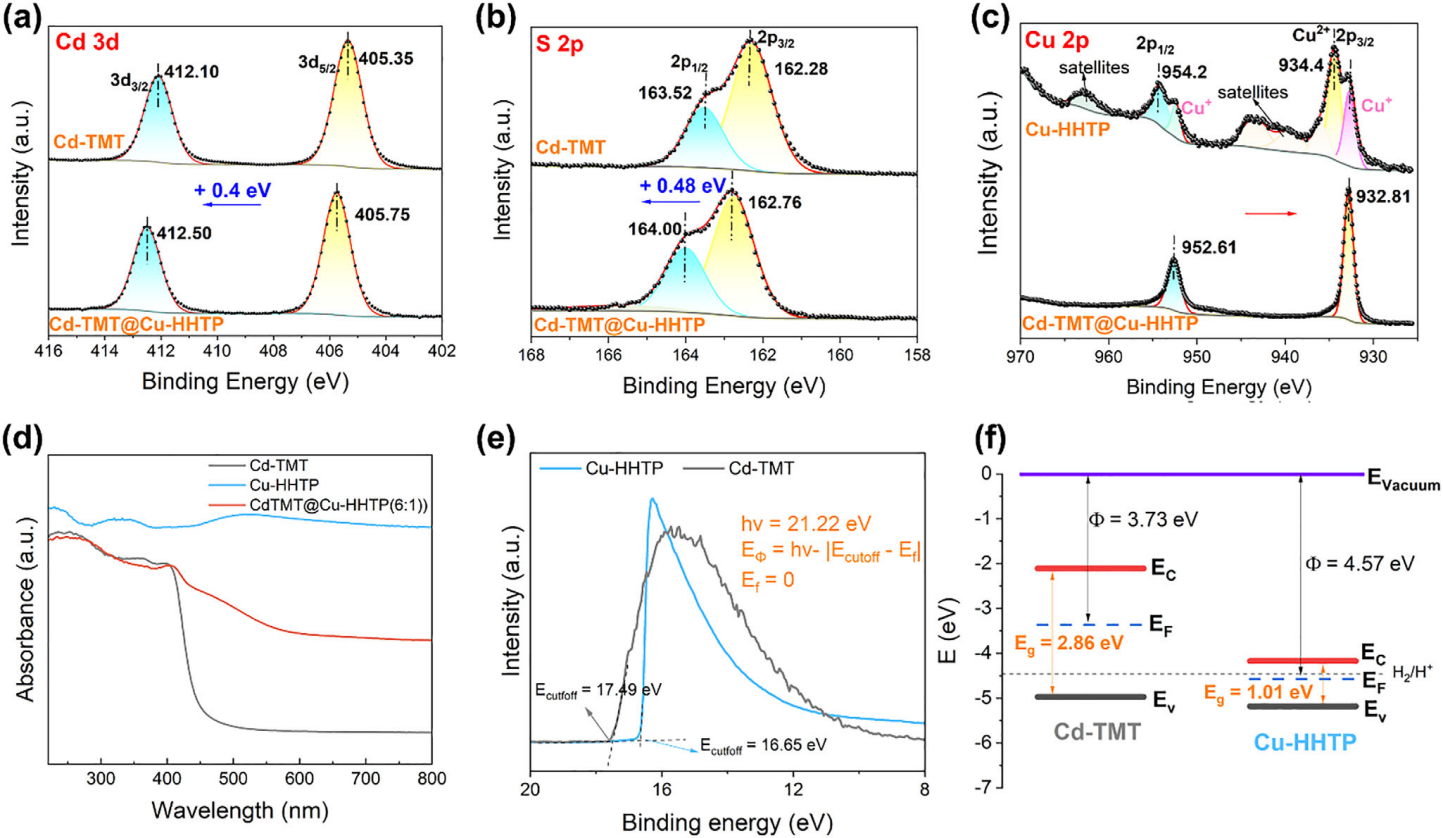
High‐resolution XPS spectra of (a) Cd 3d, (b) S 2p, and (c) Cu 2p of Cd‐TMT, Cu‐HHTP, and Cd‐TMT@Cu‐HHTP (6:1). (d) UV‐vis DRS spectra of Cd‐TMT, Cu‐HHTP, and Cd‐TMT@Cu‐HHTP. (e) UPS spectra of Cd‐TMT and Cu‐HHTP. (f) Schematic illustration of the energy band structure of Cd‐TMT and Cu‐HHTP.

X‐ray photoelectron spectroscopy (XPS) was employed to investigate charge transfer between Cd‐TMT and Cu‐HHTP within the constructed heterostructure. The full survey spectrum of Cd‐TMT@Cu‐HHTP reveals the presence of Cd, S, N, C, and O elements (Figure S5), consistent with the elemental mapping results. The high‐resolution XPS spectra of Cd 3d, S 2p, and Cu 2p are shown in Figure 4a–c. The Cd 3d spectrum of Cd‐TMT shows two main peaks at 405.35 and 412.10 eV, corresponding to 3d_5/2_ and 3d_3/2_, respectively. After integrating with Cu‐HHTP, both peaks shift positively by ∼0.4 eV, indicating a decreased electron density around Cd centers. A similar positive shift is also observed in the S 2p spectrum, suggesting an overall electron depletion in the Cd‐TMT framework. In contrast, the Cu 2p spectrum shows an opposite trend. For pristine Cu‐HHTP, the Cu 2p region can be deconvoluted into characteristic peaks of Cu^2+^ (934.4 and 954.2 eV) and Cu^+^ (932.6 and 952.4 eV), indicating the coexistence of mixed valence states, with Cu^2+^ as the dominant species. This is consistent with the previous reference [35, 36]. Notably, in Cd‐TMT@Cu‐HHTP, the Cu 2p spectrum is dominated by two sharp peaks located at ∼932.81 and 952.61 eV, corresponding to Cu^+^ species, suggesting a significant reduction of Cu^2+^ to Cu^+^. These results confirm strong interfacial electronic coupling in Cd‐TMT@Cu‐HHTP, driven by interfacial charge transfer between Cd‐TMT and Cu‐HHTP.

The optical properties of the samples were investigated by solid‐state UV‐vis diffuse reflectance spectroscopy (DRS), as shown in Figure 4d. Pristine Cd‐TMT exhibits an absorption edge at ∼450 nm, while Cu‐HHTP displays broad and intense absorption across the UV‐visible region, which can be attributed to its extended *π*‐*d* conjugation. Upon integration with Cu‐HHTP, the composite shows a significantly red‐shifted and broadened absorption edge, indicating enhanced light‐harvesting capability. Based on the corresponding Tauc plots (Figure S6), the optical band gaps (E_g_) of Cd‐TMT and Cu‐HHTP are estimated to be 2.86 and 1.01 eV, respectively. Notably, the experimentally determined band gap of Cd‐TMT is larger than the DFT‐derived values, which can be attributed to the intrinsic band gap underestimation of conventional DFT methods due to self‐interaction errors [37]. To further elucidate the band structures, ultraviolet photoelectron spectroscopy (UPS) measurements were performed to determine the work functions and valence band positions. As shown in Figure 4e, the work functions (*ϕ*) of Cd‐TMT and Cu‐HHTP were calculated to be 3.73 and 4.57 eV, respectively. The valence band spectra reveal that the energy differences between the valence band maximum (E_V_) and the Fermi level (E_F_) are 1.60 eV for Cd‐TMT and 0.61 eV for Cu‐HHTP (Figure S7). Accordingly, the conduction band positions (E_C_) are determined to be −2.11 and −4.17 eV based on the relationship E_g_ = E_C_—E_V_. The resulting band structures are illustrated in Figure 4f.

**FIGURE 5:**
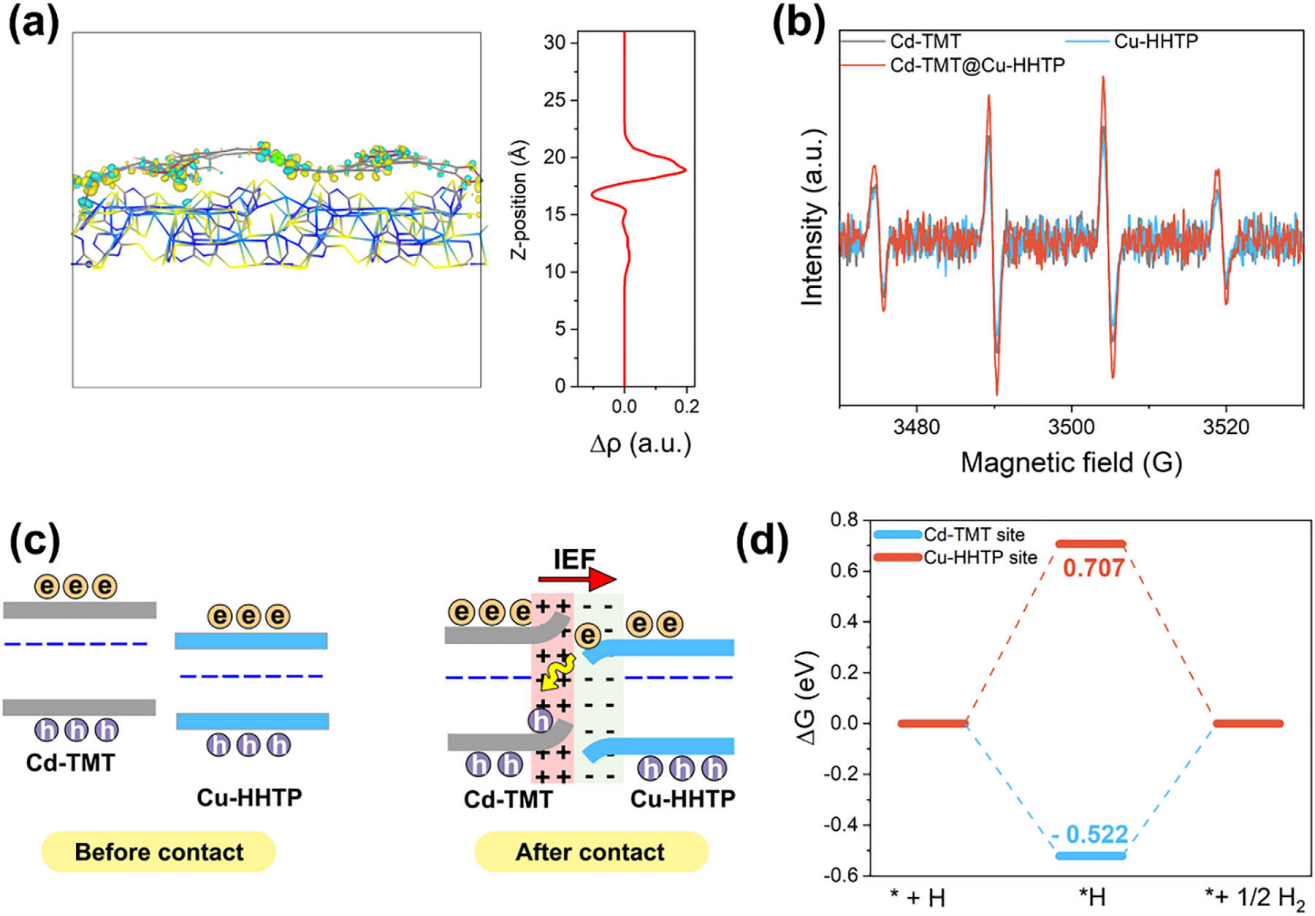
(a) Charge density difference and planar‐averaged electron density difference along with the *Z*‐direction of Cd‐TMT@Cu‐HHTP. (b) ESR spectra of DMPO‐·OH under light over Cd‐TMT, Cu‐HHTP, and Cd‐TMT@Cu‐HHTP. (c) Energy band scheme before and after the contact of Cd‐TMT and Cu‐HHTP. (d) Gibbs free energy diagrams for hydrogen adsorption on the Cd‐TMT and Cu‐HHTP sites of the heterostructure.

The charge carrier behavior was further elucidated by DFT calculations and electron spin resonance (ESR) measurements. As shown in Figure 5a, the planar‐averaged charge density difference along the *Z*‐direction clearly reveals electron transfer from Cd‐TMT to Cu‐HHTP, in good agreement with the XPS and UPS results. Under light irradiation, the Cd‐TMT@Cu‐HHTP heterojunction exhibits a stronger ·OH ESR signal than either pristine Cd‐TMT or Cu‐HHTP (Figure 5b), indicating the effective preservation of highly oxidative holes after interfacial charge transfer. Together with the DFT, XPS, and UPS results, these findings indicate the formation of an S‐scheme heterostructure (Figure 5c,d). The interfacial charge redistribution induces band bending and establishes an internal electric field (IEF) from Cd‐TMT to Cu‐HHTP. Driven by this IEF, photogenerated electrons in Cu‐HHTP migrate and recombine with holes in Cd‐TMT at the interface, thereby suppressing charge recombination while preserving highly reducing electrons in Cd‐TMT for photocatalytic hydrogen evolution [38, 39, 40, 41]. DFT calculations of the Gibbs free energy further reveal that hydrogen adsorption on Cd‐TMT is thermodynamically more favorable than on Cu‐HHTP (Figure 5d and Figure S8). Specifically, Cd‐TMT exhibits an obviously lower |ΔG_*H_| value (0.522 eV) compared to that of Cu‐HHTP (0.707 eV), suggesting more balanced hydrogen adsorption and desorption kinetics on the Cd‐TMT surface. Therefore, the S‐scheme charge transfer ensures the accumulation of high‐energy electrons on Cd‐TMT, and the favorable hydrogen adsorption energetics identify Cd‐TMT as the dominant catalytic site. This synergistic interplay between charge separation and reaction thermodynamics accounts for the markedly enhanced photocatalytic hydrogen evolution performance of Cd‐TMT@Cu‐HHTP.

## 3 Conclusion

In summary, the crystal structure and optoelectronic properties of Cd‐TMT were systematically investigated. Benefiting from its conjugated configuration and pronounced *π–π* stacking, Cd‐TMT exhibits efficient charge separation and transport, rendering it a promising photocatalyst. By integrating the conductive Cu‐HHTP, the photocatalytic hydrogen evolution performance is significantly enhanced, delivering an average H_2_ evolution rate of 9692.83 µmol g^−1^ h^−1^. Combined experimental and theoretical results reveal that the constructed heterostructure follows an S‐scheme charge transfer mechanism, enabling the efficient preservation of highly reductive electrons in Cd‐TMT. This work provides new insights into the rational design of conjugated MOF‐based heterostructures for efficient solar‐to‐fuel conversion.

## 4 Experimental Section/Methods

### 4.1 Synthesis

#### 4.1.1 Synthesis of Cd‐TMT

The Cd‐TMT was synthesized via a co‐precipitation reaction with a little modification from the previous reference [20]. Specifically, Cd(NO_3_)_2_·4H_2_O (0.015 mol) was dissolved in 200 mL of deionized water under stirring to form solution A. Trithiocyanuric sodium (0.01 mol) was dissolved in 200 mL of water to obtain solution B. Solution B was then slowly added to solution A under vigorous stirring. The resulting mixture was aged for 24 h under mild stirring. The precipitate was then collected, washed several times with deionized water, and finally dried at 80°C overnight.

#### 4.1.2 Synthesis of Cd‐TMT@Cu‐HHTP

Cd‐TMT@Cu‐HHTP composites with different mass ratios (2:1, 4:1, 6:1, 8:1, and 12:1) were prepared by dispersing varying amounts of Cd‐TMT (22, 45, 67, 90, and 135 mg) in 8 mL of deionized water, followed by the addition of HHTP (13 mg) and Cu(OAc)_2_ (8 mg). The mixture was subjected to ultrasonic treatment for 10 min and allowed to stand for 2 h. The resulting precipitate was collected, washed repeatedly with deionized water and ethanol until the supernatant was colorless, and dried at 80°C overnight.

#### 4.1.3 Synthesis of Cu‐HHTP

Cu‐HHTP was synthesized according to a previous report [35]. HHTP (0.05 mmol) was dissolved in 3 mL of deionized water, and Cu(OAc)_2_·H_2_O (0.05 mmol) was dissolved in 2 mL of deionized water. The two solutions were then combined, and the reaction was carried out at 85°C for 2 h.

### 4.2 Characterization

Powder X‐ray diffraction patterns were recorded on the D/max‐2500 PC (Rigaku, Japan) diffractometer equipped with Cu‐K*α* radiation. Rietveld refinements were carried out using the GSAS‐II program [42], in which the scale factor, zero shift, background, peak profile, atomic coordinates, and atomic displacement parameters were refined sequentially.

Scanning electron microscopy (SEM) images were obtained using a FEI Nova Nano SEM 450 microscope. Transmission electron microscopy (TEM) analysis was performed on a JEOL‐2100F instrument operated at an accelerating voltage of 200 kV.

Raman spectra were recorded on a LabRAM Odyssey (HORIBA) spectrometer with a 532 nm excitation source.

The X‐ray photoelectron data were collected using Thermo ESCALAB 250 XI (USA) with monochromated Al K*α* radiation (1486.6 eV). The binding energies were calibrated using the C 1s peak at 284.8 eV. The UV‐vis adsorption spectra and UV‐vis DRS spectra were collected by UV‐2600i ultraviolet‐visible spectrophotometer of SHIMADZU (Japan) to analyze the band gap of the photocatalysts. The EIS, and photocurrent data were collected on a CHI 660E electrochemical workstation (Chenhua Shanghai, China).

Ultraviolet photoelectron spectroscopy (UPS) measurements were performed on a Thermo Scientific ESCALAB 250Xi using a He I light source (*hν* = 21.22 eV) with a beam spot of 2 mm. Before measurement, the samples were pressed onto copper foam substrates, and the Fermi edge was calibrated using an Au reference Steady‐state photoluminescence (PL) spectra were collected using a HORIBA Fluorolog‐3 fluorescence spectrophotometer.

The photogenerated ·OH radicals were identified via electron spin resonance (ESR) spectroscopy (EMXnano. Bruker Biospin GmbH, Rheinstetten, Germany) using 5,5‐Dimethyl‐1‐pyrroline N‐Oxide (DMPO) as the spin trapping agent.

#### 4.2.1 Photocatalytic Hydrogen Measurements

Photocatalytic hydrogen evolution was carried out in a 100 mL quartz reactor, in which 20 mg of catalyst was dispersed in 50 mL of an aqueous solution containing 0.35 M Na_2_S and 0.25 M Na_2_SO_3_ as sacrificial agents. A 300 W Xe lamp equipped with an AM 1.5G filter was used as the simulated solar light source. The evolved H_2_ was continuously quantified using an online gas chromatograph (Techcomp GC‐7920) equipped with a thermal conductivity detector (TCD).

### 4.3 DFT Calculations

The interaction region indicator (IRI) analyses were performed using ORCA 6.1 and Multiwfn [25, 43, 44]. The packed TMT ligand fragments were first fully optimized with ORCA 6.1 at the B3LYP/def2‐TZVP level, including D3(BJ) dispersion corrections to account for van der Waals interactions. The resulting structures were subsequently analyzed using Multiwfn. The three‐dimensional interaction isosurfaces and two‐dimensional scatter plots were visualized using VMD 1.9.3 [45].

The interfacial charge distribution and Gibbs free energy were further investigated by density functional theory (DFT) calculations using the CP2K/Quickstep package with the Perdew–Burke–Ernzerhof (PBE) functional [46]. A double‐ζ polarized (DZVP) MOLOPT basis set and a plane‐wave cutoff of 500 Ry were employed for all atoms. Dispersion interactions were considered using the DFT‐D3 correction scheme. Input file construction and post‐processing analyses were carried out with the Multiwfn program [44].

## Funding

This work was supported by the National Natural Science Foundation of China (No. 51872173, 22005340), Qingdao Natural Science Foundation (No. 24‐4‐4‐zrjj‐194‐jch), Natural Science Foundation of Shandong Province (No. ZR2025MS785), National Ten Thousand Talent Program for Young Top‐notch Talents, Taishan Scholars Program of Shandong Province (No. tsqn202408185), and Science and Technology Special Project of Qingdao City (No. 25‐1‐5‐cspz‐9‐nsh).

## Conflicts of Interest

The authors declare no conflicts of interest.

## Supporting information




**Supporting File**: advs76912‐sup‐0001‐SuppMat.docx.

## Data Availability

The data that support the findings of this study are available from the corresponding author upon reasonable request.
