# Supplementary material for: Robust Photocatalytic Hydrogen Evolution Over a Conjugated Metal‐Organic Framework Heterojunction
Source: Adv Sci (Weinh). 2026 Aug 3:e76912. Online ahead of print. doi: 10.1002/advs.76912 (PMC13430926; doi:10.1002/advs.76912)
Supplement: Supplementary file 1 — Supporting File: advs76912‐sup‐0001‐SuppMat.docx. [file ADVS-9999-e76912-s001.docx]

Supporting Information

Robust Photocatalytic Hydrogen Evolution Over a Conjugated Metal-Organic Framework Heterojunction

Chunzhe Wang, Yufei Shan, Ruobing Chu, Lingzhi Yang, Zhanning Liu*, Xiaoli Zhang*, Jian Tian*

**Table S1**. The optimized structural parameters of Cd-TMT.

| Element | x | y | z | Occ | Sym |
| --- | --- | --- | --- | --- | --- |
| Cd | 0.87638 | 0.51203 | 0.76357 | 1.00 | 24d |
| S | 0.29846 | 0.40282 | 0.60400 | 1.00 | 24d |
| S | 0.37377 | 0.39337 | 0.11638 | 1.00 | 24d |
| N | 0.44934 | 0.35982 | 0.49608 | 1.00 | 24d |
| N | 0.37966 | 0.26573 | 0.25382 | 1.00 | 24d |
| C | 0.33146 | 0.34420 | 0.22120 | 1.00 | 24d |
| C | 0.37485 | 0.42137 | 0.50976 | 1.00 | 24d |

Space group: *Pa*-3, a = b= c = 13.94380 Å, α = β = γ = 90°.


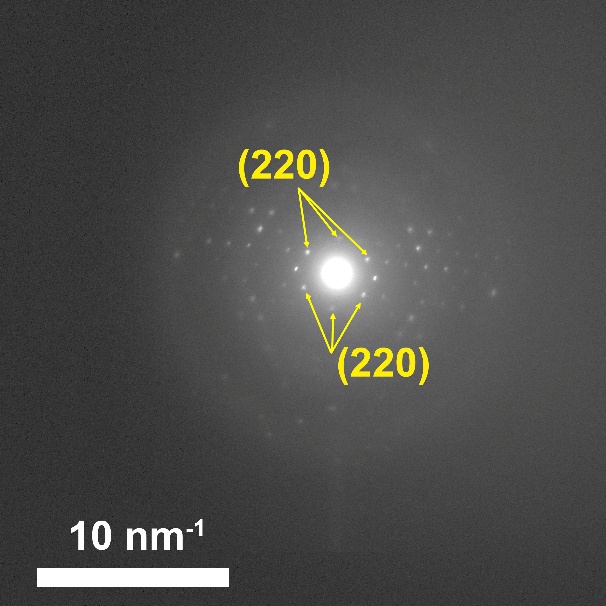


**Figure S1**. SAED pattern of Cd-TMT.


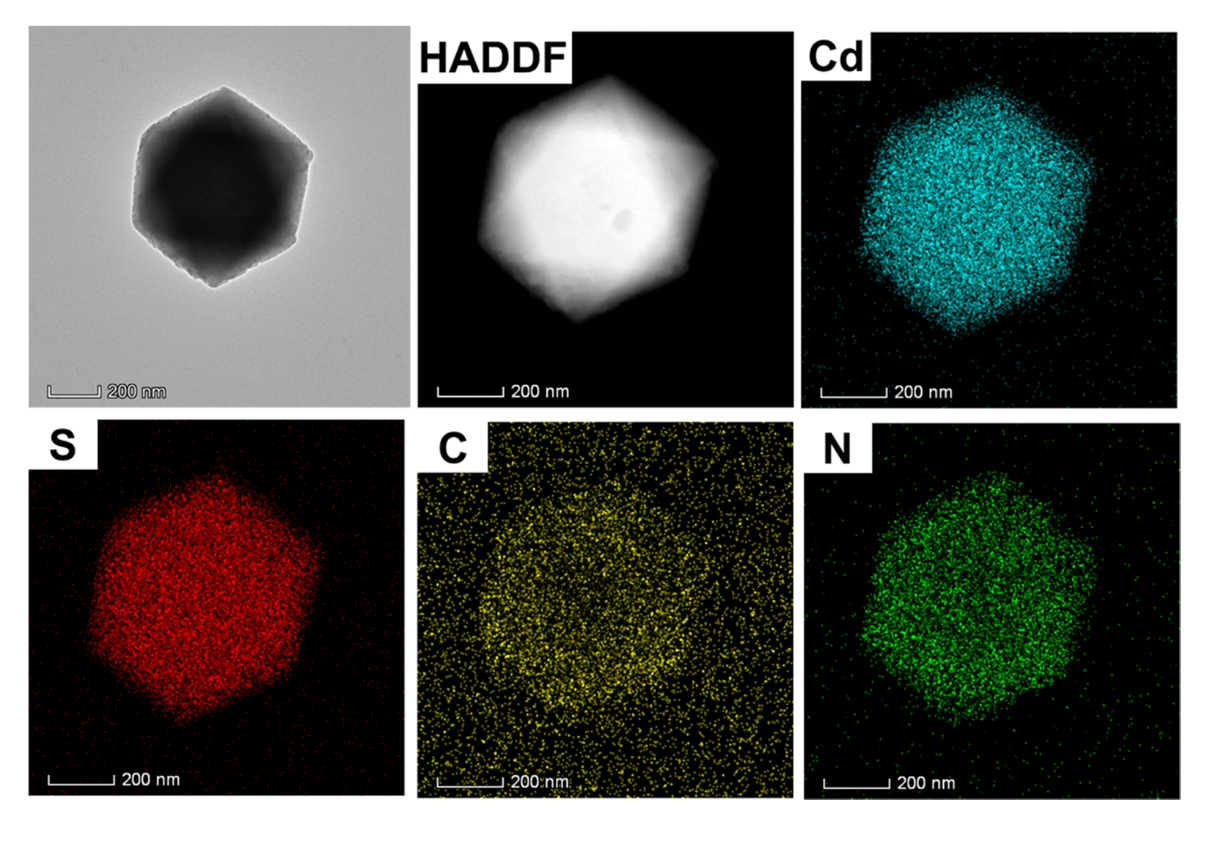


**Figure S2**. Elemental mapping images of Cd-TMT.


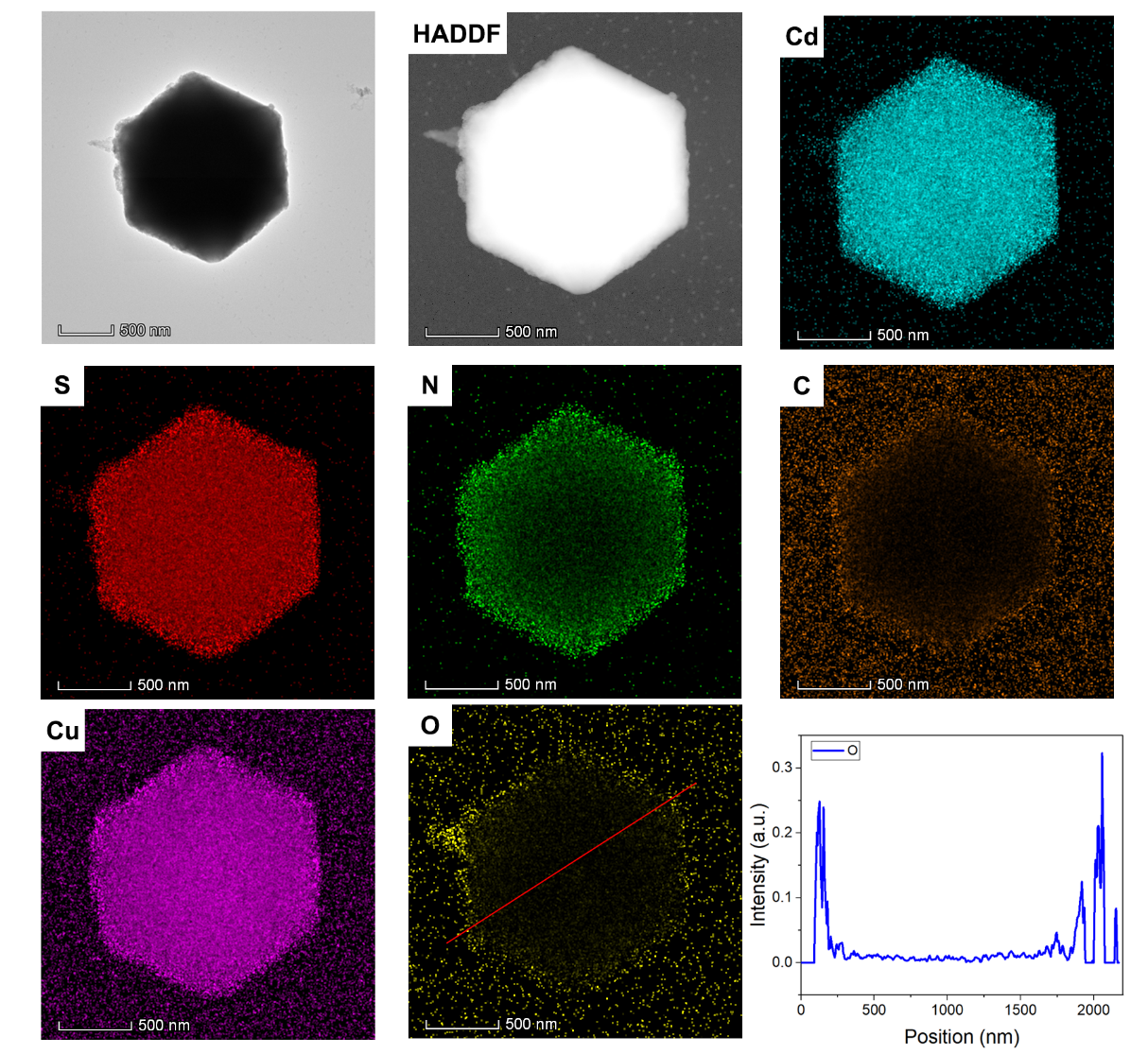


**Figure S3.** Element mapping of images of Cd-TMT@Cu-HHTP (6:1). To eliminate the influence of the Cu TEM grid, the spatial distribution was analyzed using O elemental line-scan profiles.


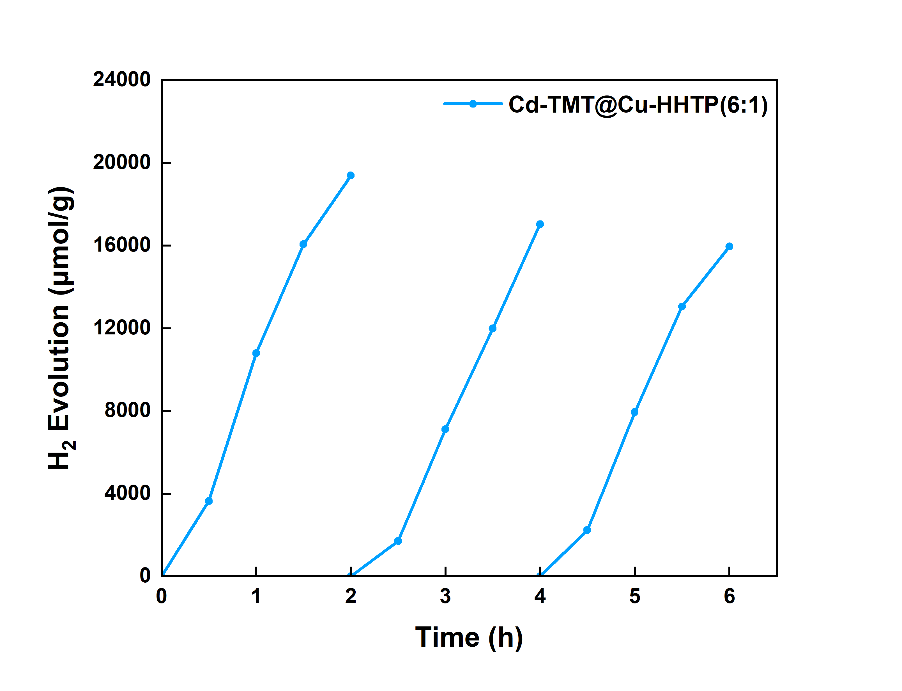


**Figure S4.** Cycling tests of photocatalytic hydrogen generation of Cd-TMT@Cu-HHTP (6:1).


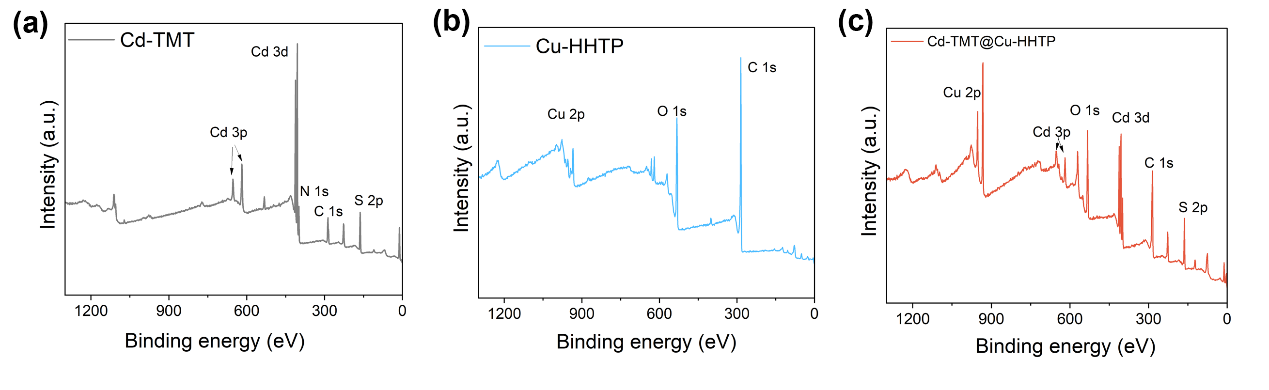


**Figure S5.** XPS survey spectra of (a) Cd-TMT, (b) Cu-HHTP, and (c) Cd-TMT@Cu-HHTP (6:1).


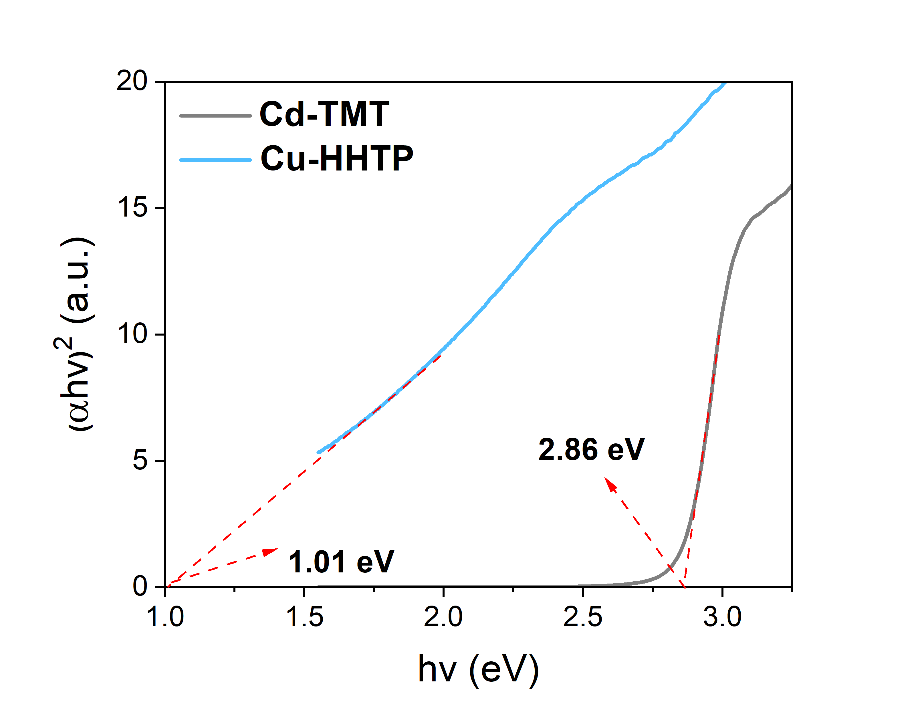


**Figure S6.** Tauc plots of Cd-TMT and Cu-HHTP.


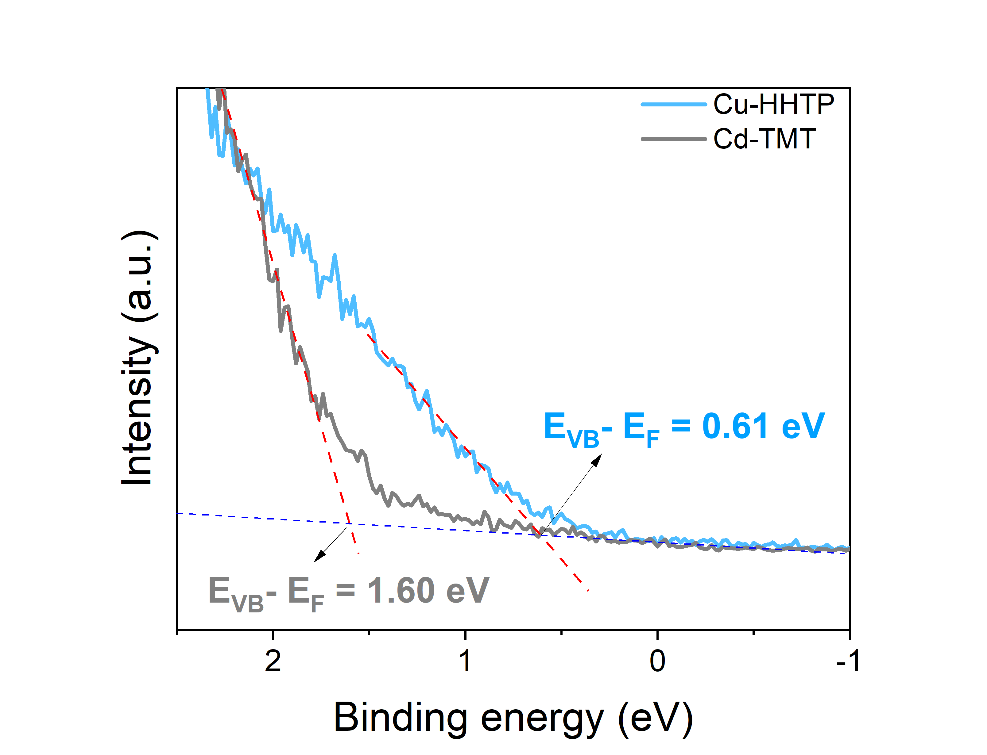


**Figure S7.** UPS spectra of Cd-TMT and Cu-HHTP at the valence band edge. Tangents are used to determine the valence band maximum.


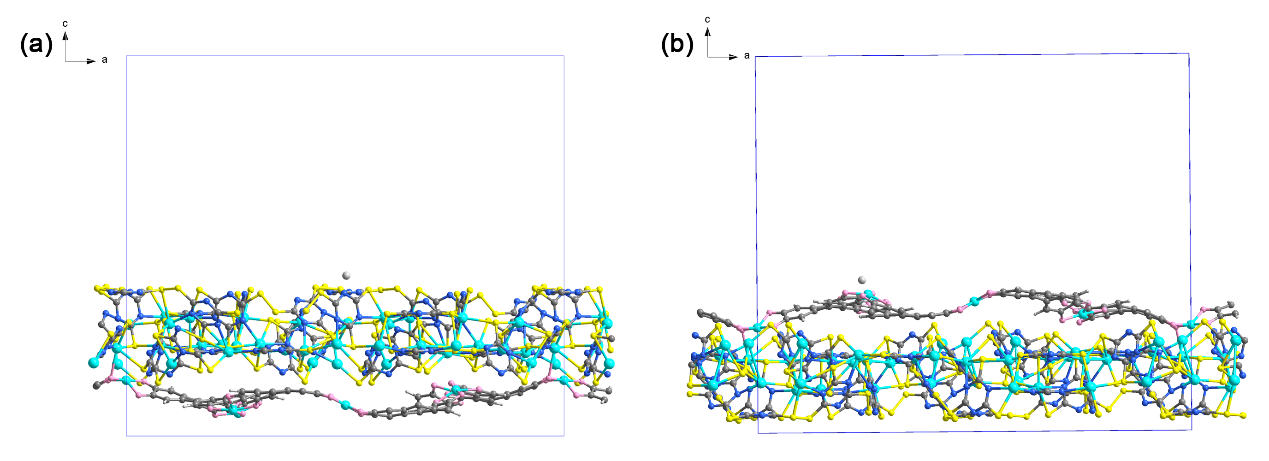


**Figure S8**. DFT-calculated structural models for the HER on (a) the Cd-TMT site and (b) the Cu-HHTP site.
